# Comparison of surgical strategies in the treatment of low-risk differentiated thyroid cancer

**DOI:** 10.1186/s12902-023-01276-8

**Published:** 2023-01-26

**Authors:** András Kiss, Balázs Szili, Bence Bakos, Richárd Ármós, Zsuzsanna Putz, Kristóf Árvai, Barbara Kocsis-Deák, Bálint Tobiás, Bernadett Balla, Henriett Pikó, Magdolna Dank, János Pál Kósa, István Takács, Péter Lakatos

**Affiliations:** Department of Internal Medicine and Oncology, Semmelweis University Faculty of Medicine, 1082 Korányi S. u. 2/a, Budapest, Hungary

**Keywords:** Thyroid cancer, Thyroid nodules, Well differentiated thryoid cancer, Surgery

## Abstract

**Context:**

Increasing diagnostic sensitivity in the detection of thyroid cancer has led to uncertainties in the optimal surgical approach of the smaller, low risk tumors. Current ATA guidelines consider lobectomy safe between 1 and 4 cm, while ETA advocates for primary total thyroidectomy to avoid reoperation, as final risk stratification is based on the histological results.

**Objective:**

Our aim was to compare the differences in outcomes that are potentially achievable with adherence to the different guidelines, and also to examine the predictive value of clinical parameters on the incidence of postoperative risk factors.

**Methods:**

We performed a retrospective cohort database analysis to identify the different surgical outcomes (based on postoperative risk factors) using ATA and ETA guidelines; the hypothetical rate of completion thyroidectomy when ATA or ETA recommends lobectomy; the accuracy of our preoperative evaluation; the utility of preoperative findings in predicting the optimal surgical strategy using binary logistic regression.

**Results:**

Out of 248 patients, 152 (ATA) and 23 (ETA) cases would have been recommended for initial lobectomy. Following the guidelines, a postoperative risk factor would have been present in 61.8, and 65.2% of the cases, respectively. Except for angioinvasion, tumor size was not a significant predictor for the presence of postoperative risk factors.

**Conclusion:**

Current pre-operative criteria are inadequate to accurately determine the extent of initial surgery and our postoperative findings verify the frequent need for completion thyroidectomy using both guidelines. As a consequence, in the absence of effective pre-operative set of criteria, we advocate primary total thyroidectomy in most cases.

## Introduction

Incidence of thyroid cancer has tripled in the last three decades, although the mortality rate of differentiated thyroid cancer (DTC) persists [1]. Overdiagnosis by the general use of novel ultrasound (US) techniques is one of the main reasons for the „TC epidemic” [2–4]. The indolent behavior [5] requires the most conservative, cost-efficient, yet well-founded and free of complication management. The most controversial issue is the surgical approach of the 1–4 cm low risk DTCs, where two main approaches exist: initial lobectomy or total thyroidectomy. Initial lobectomy may lead to a potentially lower surgical complication rate and less frequent need for thyroxine (T4) substitution. The latest guidelines of the American Thyroid Association (ATA) [5, 6], follow this approach and advocate for lobectomy in low risk DTCs between 1 to 4 cm in diameter. The final risk stratification, however, is based on the postoperative pathologic assessment (e.g. lymph node metastasis, angioinvasion, extrathyroidal invasion, multifocal disease, etc.).

After the final histology result, contralateral lobectomy is needed in certain cases. These microscopic features are less well predictable by prior clinical investigation [7]. Undiscovered contralateral foci are also needed to be considered. Thus the greatest concern about this approach is the high rate of completion thyroidectomy [8–10], with all of its disadvantages which include a second general anesthesia and hospital admission, surgical difficulties raised by scar tissue, but also psychological concerns. Furthermore, without the total/near total removal of thyroid tissue, the adjuvant I^131^ ablation and the serum thyroglobulin (Se TG) follow-up could not be achieved. Thus, the European Thyroid Association (ETA) guidelines [11] suggest a lower threshold (1 cm or above in tumor diameter) for total thyroidectomy taking into account the above detailed considerations.

In this retrospective cohort study, our aim was to compare the differences in outcomes that are potentially achievable with adherence to these different guidelines, using real-life patient data from our institution.

## Material and methods

### Patients and setting

We performed a retrospective patient database analysis. We included patients who were referred to our tertiary referral center with differentiated thyroid cancer between January 2014 and October 2018 for radioiodine treatment evaluation. Surgical indications and operation plans were established in other centers in some cases. Although surgical indication could either be clinically benign or malignant thyroid disease, our patients were selected retrospectively based on histopathology. Inclusion criteria were: final histology of DTC (any subtype of papillary, follicular or Hürthle-cell carcinoma) and total/near total removal of the thyroid gland (either in one or more steps). Patients whose thyroid gland had not been removed (near) totally were excluded from the evaluation. Cancer diagnosis for every case was based on histological data from surgical specimens, using the WHO Classification 3rd and 4th edition. Staging was based on the TNM system 7th edition. For preoperative staging, clinical investigation was used (ultrasound in all cases [most of them with FNAB], and CT, MR, PET-CT in some cases). Mostly a selective lymph node dissection was performed, but in some cases documentation was poor, and malignancy was not suspected preoperatively – resulting no lymph node dissection. No other inclusion or exclusion criteria were set.

The analyzed patient data included age, past medical and familial history (neck and thoracic irradiation, thyroid cancer in relatives), preoperative tumor characteristics, type of surgery, surgical complications and postoperative histological findings. Preoperative tumor caracteristics were: size on imaging, multifocality and/or bilateral tumor presence, lymph node (LN) and/or distant metastases, extrathyoidal extension, aggressive histologic variant with FNAB. Postoperative histological findings included: cancer type and subtype, pathological staging, angioinvasion, tumor capsule invasion, extrathyroidal extension, signs of incomplete tumor resection.

A./ We retrospectively evaluated the different outcomes that could have been achieved using surgical approaches based on the ATA and ETA guidelines and these were compared to our practice. Clinical data from all patients were re-evaluated using both of these guidelines and we established the type of surgery that would have been indicated based on the preoperative data. In cases where one or both guidelines advocated for lobectomy, the need for hypothetical reopreration was evaluated based on postoperative data.

B./ We compared our institute’s practice to both the ATA and ETA guidelines by analyzing all cases where one step total/near total thyroidectomy was performed despite one or the other association’s guideline recommending lobectomy based on the preoperative data.

We evaluated the need for completion thyroidectomy after a hypothetical lobectomy based on the available postoperative histological data.

C./ We assessed the accuracy of our preoperative evaluation in predicting the optimal surgical course when compared to postoperative histological data. Positive and negative predictive values (PPV and NPV, respectively), sensitivity and specificity were calculated. Cohen’s kappa was calculated to evaluate concordance between preoperative ATA or ETA recommendations and the histopathology based correct course of action.

We examined the utility of preoperative ultrasound and clinical findings in predicting the optimal surgical strategy. These incuded tumor diameter, age, sex, and presence of Hashimoto thyroiditis. The relevance of tumor size in predicting individual postoperative high-risk features, such as bilateral tumor, angioinvasion, extrathyroid extension, incomplete surgical resection, aggressive histology and lymph node metastasis, was also evaluated.

D./ We compared the complication rate in different types of surgeries.

### Statistical methods

Data are given as mean ± SD for continuous and frequencies for discrete variables. Between-group differences with categorical predictors and outcomes (eg. complication rates of different surgical strategies) were assessed using Chi-square and Fisher’s exact test. In cases with one or more independent scale variable (eg. predictive value of tumor diameter on surgical outcome), binary logistic regression was used to build the appropriate model. Unless otherwise noted *p*-values less than 0.05 are considered statistically significant. Analyses were conducted using IBM SPSS Statistics for Windows, Version 23.0 (IBM Corp. Released 2015 Armonk, New York, USA.).

### Ethical approval

The study was approved by Semmelweis University Regional and Institutional Committee of Science and Research Ethics (Egészségügyi Tudományos Tanács, Tudományos és Kutatásetikai Bizottság (under case number IV/4097–2/2020/EKU)). Since this retrospective cohort study was performed on an anonymous database, the informed consent was waived.

## Results

We identified 261 potential patients in our database that satisfied inclusion criteria. Two of them were excluded based on anaplastic or medullary histology. Out of the 259 patients, 76.4% were female and 23.6% were male. In 86.9% of cases papillary thyoid cancer, in 10.0% follicular and in 3.1% Hürtle cell histologic type was established. A further 11 patients were excluded from further analysis as they underwent lobectomy without completion. However, 248 met the inclusion criteria, where applicable, 259 well-differentiated thyroid cancer patient were included in the calculations, e.g. post-lobectomy complications.

A./ From the final 248 patients, 152 (61.3%) and 23 (9.3%) cases would have been recommended for initial lobectomy according to ATA and ETA, respectively. Based on postoperative histological data, these hypothetical lobectomies would have been inadequate in 94 (61.8%) and 15 (65.2%) cases (ATA and ETA, respectively) leading to completion thyroidectomy. Following a hypothetical lobectomy, the most common indications for repeated surgery would have been: previously unknown lymph node metastases, incomplete surgical resection (R1 resection or positive surgical margin), extrathyroidal or vascular invasion and bilateral tumor. Bilateral tumor would have been discovered later, so a later reoperation would have had to be performed.

Among our patients ATA would have recommended total thyroidectomy in 38.7% of the cases. The most frequent indication for primary total thyroidectomy according to ATA guidelines was tumor size above 4 cm. In contrast to this, 90.7% of our patients were candidates for total thyroidectomy using the ETA guideline. In cases where the guidelines advocated for initial (near) total thyroidectomy, this decision would have been justified in 81 of 96 (84.4%) (ATA) and 161 of 225 (71.6%) (ETA) of cases. In other words, we found one or more histological risk factors in 84.4 and 71.6% of the surgical specimens in these patients.

There was a statistically significant difference between the ATA and ETA guidelines in the frequency of recommending initial lobectomy. The hypothetical need for subsequent reoperation, however, did not appear to be different – with the absolute number of reoperations being much lower (15 vs. 94 of 259 patients). In cases where the individual guidelines called for initial thyroidectomy, the ATA guideline had a higher positive predictive value in indentifying cases with high risk histology. Data is shown in detail in Table 1.

**Table 1:** Hypothetical indications and outcomes in patients whose thyroid gland was removed in one or two steps, according to the 2 main social guidelines, based on the pre-and postoperative findings

	ATA		ETA		*p*-value
	N	%	N	%	
Lobectomy	152	61,30%	23	9,30%	< 0,001
Total thyroidectomy	96	38,70%	225	90,70%	
Lobectomy completion needed	94	61,80%	15	65,20%	0,756
Lobectomy only sufficient	58	38,20%	8	34,80%	
After total thyroidectomy					
risk factor(s) positive	81	84,40%	161	71,60%	0,021
risk factor negative	15	15,60%	64	28,40%	

Compared to ATA recommendations, we performed primary total thyroidectomies more frequently (38.7% vs. 73.8%). However, ETA would have recommended even more one step total thyroidectomies (90.7% vs. 73.8%). Lobectomies were followed by a second surgery in most of our cases (91.2%).

If ATA would have recommended total thyroidectomy and we followed this recommendation in 81.3% of these cases, this was proved to be eligible in 84.4% of cases. If ATA would have recommended lobectomy and we have performed accordingly, at least one risk factor was shown in the histology result in 69.0% of cases. In cases where ETA would have recommended total thyroidectomy and in 77.8% of cases we have done so, resulting a 73.7% postoperative risk factor ratio – compared to the 71.6% if we had proceeded entirely according to ETA.. Postoperative risk factor ratio is calculated by number of cases where at least 1 postoperative risk factor was present devided by number of all cases. Interestingly, if ETA indicates lobectomy, and we have performed the surgery accordingly (39.1%), the ratio of the postoperative risk factor was found to be 88.9%.

Indicators of ATA have turned out to be superior to ours: positive predictive values were 84.4% vs. 71.7%; negative predictive values were 38.2% vs. 32.3%; false positive rates were 15.6% vs. 28.3%; false negative rates were 61.8% vs. 67.7%, respectively.

B./ Primary total thyroidectomy was undertaken at our institute in 110 of the 152 cases where the ATA guideline would have recommended lobectomy. Postoperative histological results validated this approach in 60.0% of cases.. The identified risk factors are shown in Table 2. The distribution of these risk factors among the 66 of our patients who underwent primary total thyroidectomy despite the ATA guideline recommending lobectomy was the following: 38 patients had 1 risk factor, 15, 10 and 3 patients presented with 2, 3 and 4 risk factors respectively. Undiscovered lymph node metastasis would have been the leading cause (47.0%) of consequent reoperation had these patients undergone primary lobectomy. Other factors were incomplete surgical resection (34.8%) and extrathyroidal extension (27.3%, mainly microscopic). Furthermore, in 12 of 110 cases (10.9%), a contralateral tumor would have remained undiscovered. Two, 3 and 4 concurrent risk factors were discovered in 22.7, 15.2 and 4.6% of these patients, respectively.

**Table 2:** Frequency of postoperative risk factors in those, who reasonably underwent total thyoroidectomy

Postoperative risk factors in *n* = 66 patients	Men (*n* = 16)		Women (*n* = 50)		Total (*n* = 66)	
Lymph node metastasis	8	50,0%	23	46,0%	31	47,0%
Positive surgical margin	6	37,5%	17	34,0%	23	34,8%
Extrathyroidal extension	4	25,0%	14	28,0%	18	27,3%
Angioinvasion	4	25,0%	13	26,0%	17	25,8%
Bilateral tumor	5	31,3%	7	14,0%	12	18,2%
Aggressive variant	1	6,3%	8	16,0%	9	13,6%
Total	28	25,4%	82	74,6%	110	100,0%

Total thyroidectomy was performed at our institute in 14 of the 23 cases where the ETA guideline would have advocated for lobectomy. This decision was confirmed by postoperative risk factor assessment in half of the cases. Of these, incomplete surgical resection and previously undiscovered lymph node metastases were once again the most common findings (71.4 and 42.9% respectively).

C./ We compared the accuracy of preoperative clinical work-up (US and FNA) in predicting postoperative histological findings. While specificity and PPV were mostly acceptable (79.5–99.1% and 44.4–100.0% respectively), sensitivity was uniformly low in all areas incuding prediction of lymph node metastasis (48.9%), identifying extrathyroidal (5.9%) or bilateral tumor (32.0%) presence, or establishing the presence of aggressive tumor variants (28%). Cohen’s kappa was 0.04 and 0.20 for ETA and ATA criteria respectively. These values reflect the low agreement between pre- and postoperative findings with the modest superiority for ATA criteria.

We examined the predictive value of preoperative clinical parameters on the type of surgical intervention in the subgroup of cases where primary total thyroidectomy was performed at our institute despite the ATA guideline advocating for initial lobectomy (*n* = 110). Of the preoperative parameters examined, only tumour size had a significantly positive (*p* = 0.001) and patient age a nearly significantly negative (*p* = 0.057) effect on the presumed need for total thyroidectomy if the ATA guideline had been followed. Model fit was moderate (*R*^2^ = 0.20 (Nagelkerke)). Results from the logistic regression are presented in Table 3.

**Table 3:** Effect of preoperative clinical parameters on disease severity defined by the histologically verified need for total thyroidectomy. *R*^2^ = 0.20 (Nagelkerke). See text for details

	OR	95% CI for Exp(B)	p
**Nodule size (mm)**	1.098	1.037–1.161	**0.001**
**Patient age (years)**	0.972	0.944–1.001	0.057
**Gender**	1.002	0.335–2.995	0.998
**Hashimoto (Y/N)**	0.574	0.225–1.466	0.246

We examined the value of tumor size in predicting individual postoperative risk factors using data from all of our patients (*n* = 259). Results from the logistic regression are shown in Table 4. After adjustment for multiple testing, preoperative tumor size significantly correlated with the presence of vascular invasion. There was a near significant association with incomplete surgical resection. However, the size of the primary malignancy, was not a significant predictor for the presence of a contralateral tumor, aggressive histology, extrathyroidal extension or undiagnosed lymph node metastasis.

**Table 4:** The effect of tumor size (mm) on the presence individual postoperative risk factors. After adjustment for multiple testing using the Bonferroni method, *p* values < 0,008 are considered statistically significant

Outcome	OR	95% CI for Exp(B)	p
Contralateral tumor	0.994	0.970–1.019	0.653
Aggressive histology	1.014	0.987–1.042	0.307
Extrathyroidal extension	1.019	0.996–1.042	0.115
Positive surgical margins	1.030	0.007–1.052	0.009
Lymph node metastasis	1.001	0.982–1.021	0.893
**Vascular invasion**	**1.047**	**1.023–1.071**	**0.000087**

D./ From the pool of all 259 patients with DTC, 38 (14.7%) patients suffered a total of 44 sugical complications. Amongst these, hypocalcaemia and temporary, unilateral recurrent laryngeal nerve palsy were the most common. Complication rate was higher with primary total thyroidectomy (15.2%) vs. lobectomy (4.4%) (*p* = 0.02).

We also assessed complication rates separately in the subgroup of cases where total thyroidectomy had been performed and its need was retrospectively validated by postoperative histological findings (*n* = 173). Both the overall rate of complications and the frequency of specific complications were similar in patients who underwent total thyroidectomy in one step versus those who had lobectomy followed by completion thyroidectomy (18.8% vs. 19.5%, Exact *p*-value = 1.00).

## Discussion

In the present study we compared the outcomes of DTC management using our institute’s protocol and the potential outcomes that could have been achieved with strict adherence to the ATA or ETA guidelines. We evaluated the predictive power of preoperative ultrasound, cytological, and other clinical features in this context. Following these recommendations would have led to the same relative reoperation rate (absolute number of reoperations would have been much higher according ATA guidelines and initial lobectomy would have been more frequent), and unrecognized lymph node metastases would have been the most common reason for completing thyroidectomies. Tumor size was not associated with the incidence of postoperative risk factors, excluding angioinvasion. Neither the tumor size, nor any other clinical data could predict adequately the optimal surgical approach. Our postoperative risk factor positivity ratio was very similar to that of ETA – this was found to be more eligible in lobectomies, but less in total thyroidectomies. However, predictive values of ATA were superior to our practice.

This uncertainty of preoperative studies argues for a more radical surgical approach. A high number of primary total thyroidectomies were performed at our institution despite more lenient ATA recommendations. We advocacte for near total thyroidectomy in more cases of thyroid cancer since the false negative ratio above 60% is considered to be inacceptable with particular regard to bilateral tumors.

Our postoperative risk factor positivity ratio was very similar to that of ETA – this was found to be more eligible in lobectomies, but less in total thyroidectomies. In a similar paper, published by DiMarco et al [8], from the pool of 275 FNAB-confirmed Bethesda V-VI cytology papillary thyroid cancers between 1 and 4 cm in size that were candidates for lobectomy according to ATA, 92.0% underwent complete thyroidectomy and the reoperation rate would have been 42.5%. In contrast to our study, angioinvasion (30.8%) and tumor invasion (23.9%) were the most frequent reason for the hypothetical completion thyroidectomy. Among our patients according to both ATA or ETA, the reoperation rate would have been nearly 50%. Neither bivarible analysis, nor multivariable logistic regression model found a significant predictive value for completion thyroidectomy among age, sex, tumour size, family history and FNAB result. Also in Kjolhuff’s work [10], of the 149 low-risk differentiated thyroid cancer between 1 and 4 cm eligible for lobectomy according to ATA, 19.5% would have had to be reoperated to undergo adjuvant radioiodine treatment regarding the criteria set by ATA. If the relative indications (that are commonly used indications for radioiodine treatment (RAI) although not included in ATA guideline) are considered, 49.0% would have had to be reoperated.

ATA guidelines were based on findings that the tumor size of 1–4 cm in diameter alone should not influence the extent of surgery as it is not associated with survival [12, 13]. However, confounding factors, such as aggressive variants, lymph node metastasis, positive surgical margin, male gender, lack of RAI do affect survival, and thus, those might have an impact on the decision making regarding the extent of surgery. Unfortunately, the preoperative assessment of these factors is markedly unreliable.

Our data suggest that growing tumor size is associated with an increasing risk for angioinvasion and positive surgical margin, however, the risk for bilateral tumor, lymph node metastasis, aggressive variants, and extrathyroidal extension do not decrease with smaller tumor diameter, thus, suggesting the need for total thyroidectomy.

As the most permissive strategy, in an observational trial for following papillary thyroid microcarcinomas [14] it was found that follow-up alone could be a reasonable approach in patients without any unfavorable clinical or histological features. Thirtytwo percent of the patients in the observational group had undergone surgery, and the rate of newly identified lymph node metastasis was increasing during the observational period. Other studies have shown, that the recurrence rate is lower when total thyroidectomy is performed [15]. Based on the aforementioned data, the more frequent consideration of primary total thyroidectmy over lobectomy might be advised. Psychological factors affecting patient preference should also be taken into consideration. In our practice patients usually prefer primary complete thyroidectomy and the higher chance of becoming completely tumor-free that is associated with this approach.

Lobectomy undoubtedly has a lower complication rate than total thyroidectomy. In our database, there were no difference between the adverse event rates in one- or two-step thyroidectomy groups. Nevertheless, the double anesthesia and hospital admission certainly carry additional risks and costs. Thus, if there is no difference in terms of complications as it is demonstrated by our data, primary total strumectomy could be more beneficial for the patients.

Although the same oncological outcome could be achieved by completion thyroidectomy, leaving undetected contralateral cancer foci, a second hospital admission, general anesthesia, more absence from work and more inconvenience could be prevented. RAI treatment is aimed to decrease the recurrence rate in cases where unfavorable clinicopathological prognostic factors (high-risk for recurrence) are detected, and also to cure recurrence or metastasis [16, 17]. Our findings indicate that at present it is not possible to determine these factors accurately without the complete removal of the thyroid gland. Furthermore, in cases of postoperative upstaging after lobectomy, a second completion strumectomy will be necessary, in order to perform a radioiodine therapy. The benefits of serum thyroglobulin (TG) and anti-thyroglobulin antibody (aTG) monitoring could only be utilized following total thyroidectomy and RAI treatment.

To determine the optimal surgical approach, and to decrease the need for unnecessary thyroidectomies, a new set of criteria should be established – but until then, we suggest the more frequent application of primary total thyroidectomy. Appending previously established predictive models with new features such as genetic and epigenetic data from FNA samples is one of the newly emerging promising methods [18–22].

Our study has several advantages which include: a single institution of patient care with uniform diagnostic and therapeutic procedures, high number of cases per year, well documented pre and post-op factors which all lead to well reconstructable cases. Among the limitations of our study, we have to mention its retrospective setting. Due to the short follow-up time we cannot present survival data which is a cornerstone of cancer studies. The high ratio of patients who underwent primary total thyroidectomy can decrease the statistical power when comparing outcomes of one- vs. two-step total thyroidectomies. Exclusion of patients submitted to lobectomy alone may be a source of selection bias. Sonographic assessment was provided by multiple observers at different hospitals on different ultrasound machines. No data on interobserver variability could be collected and no clear definition of sonographic extrathyroidal extension could be adopted. The judgement on extrathyroidal extension was solely on the single ultrasound observer. In our database lymph node metastases could include micrometastasis also (as some pathologic reports did not describe the size of the metastasis), and no data on the actual number of clinically relevant metastases was available. Cost-effectiveness could not be assessed and the severity and the duration of complications are not well documented. The reconstruction may not be impeccable: the hypothetical adherence to different guidelines which we tried to model does not take into account all the detailed information that is evaluated in real life clinical scenarios.

In conclusion, we may confirm that the decision to perform total thyroidectomy instead of lobectomy, despite ATA or ETA recommendations for the latter, was a posteriori warranted in the majority of our patients. It is evident that current pre-operative criteria are inadequate to accurately determine the extent of initial surgery since our postoperative findings suggest frequent need for completion thyroidectomy. ETA has stricter criteria for lobectomies, so that the need for a second surgery is probably less frequent, however, the number of unnecessary total thyroidectomies are proportionately higher with this approach. Predicting the need for a total thyroidectomy is only one aspect of the recommendations, and we chose this variable for its ease of evaluation. We do not intend to state whether one guideline is superior to the other, but only to highlight the unreliability of preoperative measurements in general. Since the rate of reoperation would have been similar following both the ATA and the ETA guidelines and there is no reliable predictive parameter available at present to determine the proper type of surgery, more frequent primary total thyroidectomy might be suggested until effective pre-operative set of criteria will be established.

## Data Availability

The datasets used and/or analysed during the current study are available from the corresponding author on reasonable request.
